# Evaluation of left atrial and ventricular remodeling in atrial fibrillation subtype by using speckle tracking echocardiography

**DOI:** 10.3389/fcvm.2023.1208577

**Published:** 2023-08-10

**Authors:** Shirui Lu, Hongyun Liu, Jie Sun, Jun Zhang, Li Li, Qiaoying Tang, Yani Liu, Youbin Deng

**Affiliations:** Department of Medical Ultrasound, Tongji Hospital, Tongji Medical College, Huazhong University of Science and Technology, Wuhan, China

**Keywords:** atrial fibrillation, speckle tracking echocardiography, left atrial, left ventricular, strain, volume

## Abstract

**Background:**

Atrial fibrillation (AF) is associated with cardiac structural and functional remodeling. We investigated the left atrial (LA) and left ventricular (LV) changes in AF subtypes by using two-dimensional echocardiography strain techniques.

**Methods:**

The study population consisted of 102 subjects with sinus rhythm (control group) and 463 patients with AF, among which 284 patients had paroxysmal AF (PAF) and 179 patients had persistent AF (PerAF). A speckle tracking automatic functional imaging software was used to perform the strain analysis.

**Results:**

Patients with AF had dilated LA maximum and minimum volume, decreased LA reservoir strain, lower LV ejection fraction (LVEF), and impaired global longitudinal strain (GLS) compared to those of the sinus rhythm control group. In patients with PerAF, the LA maximum and minimum volumes were larger, and the LA reservoir strain [PAF vs. PerAF, 28% (21,33) vs. 19% (14, 28), *P* < 0.05], LVEF, and absolute GLS value (PAF vs. PerAF, −16.9 ± 3.3 vs. −14.1 ± 3.5%) were lower than those in patients with PAF. Patients with AF regardless of LA enlargement had decreased LA reservoir strain and lower LVEF and absolute GLS value than those in the sinus rhythm control group.

**Conclusion:**

Compared with those with normal sinus rhythm, patients with AF had dilated LA volume and impaired LA function, which were further worsened in patients with PerAF than those in patients with PAF. LA functional impairment occurred before LA enlargement. Left atrioventricular remodeling happened across different stages of AF development.

## Graphical Abstract

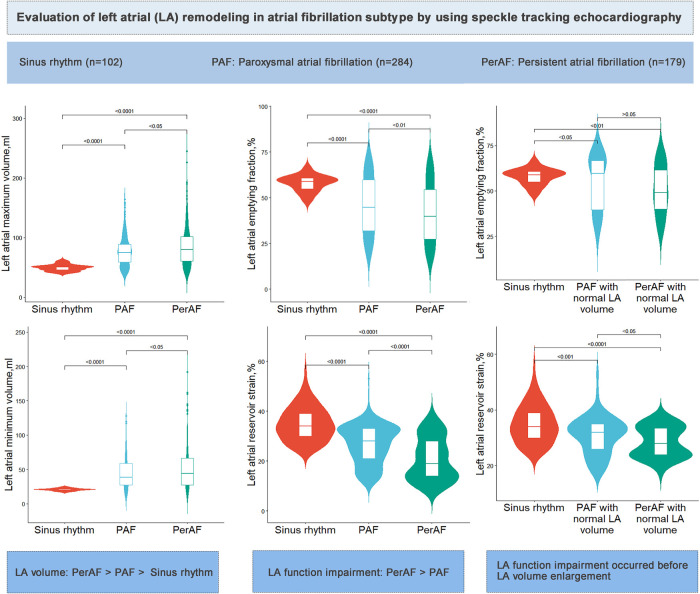

## 1. Introduction

Atrial fibrillation (AF) is a common cardiac arrhythmia that is becoming increasingly prevalent due to the aging population (1, 2). Despite efforts to understand its pathophysiology and improve treatments, identifying the underlying causes of AF in individual patients remains challenging (3, 4).

Atrial remodeling is a critical feature of AF, and speckle tracking echocardiography has emerged as an excellent approach for evaluating this process (5, 6). This imaging modality provides a non-invasive assessment of left atrial (LA) strain, which is inversely related to LA wall fibrosis and AF burden (6, 7). LA mechanics differ between AF subtypes of paroxysmal AF (PAF) and persistent AF (PerAF), and these characteristics influence the clinical interpretation of these measures (8). According to Kuppahally's report (9), PerAF had more fibrosis and lower midseptal and midlateral LA strains than PAF. However, only minimal differences in LA remodeling were found between PAF and PerAF in another study (7). Therefore, the differences in LA remodeling between AF subtypes remain debatable. In addition, LA size and function may vary at different stages of AF development. Kojima et al. (10) found that LA functional impairment was observed before LA enlargement in patients with PAF. However, this finding was based on traditional velocity vector imaging and in a relatively small number of subjects, particularly in patients with PAF. There is still a lack of enough evidence on the relationship between LA structural and functional remodeling.

In addition to irregular atrial electrical activity, AF is also characterized by generating irregular activations of the ventricle (11). Mechanical LA remodeling can further damage the active contribution to ventricular filling, resulting in reduced LV function (12). It has been reported that immediate hemodynamic changes caused by AF may contribute to decreased cardiac output and acute heart failure (13). A recent study found that patients with PerAF had significantly reduced LV ejection fraction (LVEF) than that in patients with PAF (8). Although global longitudinal strain (GLS) is a widely used LV strain parameter that provides prognostic information (14), a few studies have externally validated GLS in AF patients.

Thus, this study aimed to evaluate the AF-induced changes in LA mechanics, clarify the association of LA structural and functional remodeling in AF subtypes, and explore the left atrioventricular functional coupling across different stages of AF progression by using speckle tracking echocardiography.

## 2. Methods

### 2.1. Study population

From October 2021 to September 2022, we conducted a prospective study, which enrolled 527 patients diagnosed with AF and 102 healthy subjects with sinus rhythm. The determination of AF subtype and sinus rhythm was based on the 2020 European Society of Cardiology (ESC) guidelines. A standard 12-lead ECG recording or a single-lead ECG tracing of ≥30 s, showing heart rhythm with no discernible repeating P waves and irregular RR intervals (when atrioventricular conduction is not impaired), is diagnostic of clinical AF. PAF was defined as terminated spontaneously or with intervention within 7 days of onset. PerAF was defined as continuously sustained beyond 7 days, with episodes terminated by cardioversion (drugs or electrical cardioversion) after ≥7 days (1). The exclusion criteria comprised coronary artery disease (*n* = 26), organic valvular disease (*n* = 5), LVEF < 40% (*n* = 9), previous cardiac surgery (*n* = 6), other heart diseases or other serious non-cardiac diseases (*n* = 11), and suboptimal echocardiographic image quality (*n* = 7). After the application of the exclusion criteria, a total of 463 patients with AF were included in the analysis, among which 284 patients had PAF and 179 patients had PerAF. The Tongji Hospital Ethics Committee approved the study with approval number TJ-IRB20220621, and all participants provided their informed consent before participating in the study.

### 2.2. Clinical data

At the initiation of the study, we obtained baseline characteristics and clinical data for all participants. Heart rate was determined based on the findings of the standard 12-lead electrocardiogram. Symptoms were scored by European Heart Rhythm Association (EHRA) class (1). We calculated the CHA2DS2-VASc score based on clinical data (1).

### 2.3. Conventional transthoracic echocardiography

Transthoracic echocardiography was performed using GE Vivid E95 ultrasound equipment (GE Vingmed Ultrasound, Horten, Norway) with an M5Sc transducer (1.7–3.3 MHz) and a frame rate of 70–80 frame/s. According to the prevailing recommendations, M-mode, two-dimensional, color, pulsed, and continuous-wave Doppler data were acquired on standard views adjusting depth, sector width, and gain settings, as required. For participants with sinus rhythm, three cardiac cycles were stored for each image, while for those with AF, at least 10 were saved. All echocardiographic parameters were analyzed with an index beat (preceding RR/pre-preceding RR close to 1) in AF cases, as recommended (15). The internal diameters were measured in accordance with the quantitative method suggested by the American Society of Echocardiography (16). LV relative wall thickness (RWT) was calculated as RWT = 2 × LV posterior wall thickness/LV end-diastolic dimension. LV volumes, LVEF, and LA volumes were measured using the biplane Simpson method. All images were digitally stored for offline analysis.

### 2.4. Speckle tracking automatic functional imaging

Strain analysis was conducted using a commercial speckle tracking automatic functional imaging (AFI) software (EchoPAC version 2.4, GE Vingmed Ultrasound) (17). This software automatically tracked frame-to-frame speckle changes in two-dimensional images to assess LA and LV strain.

**Figure 1:**
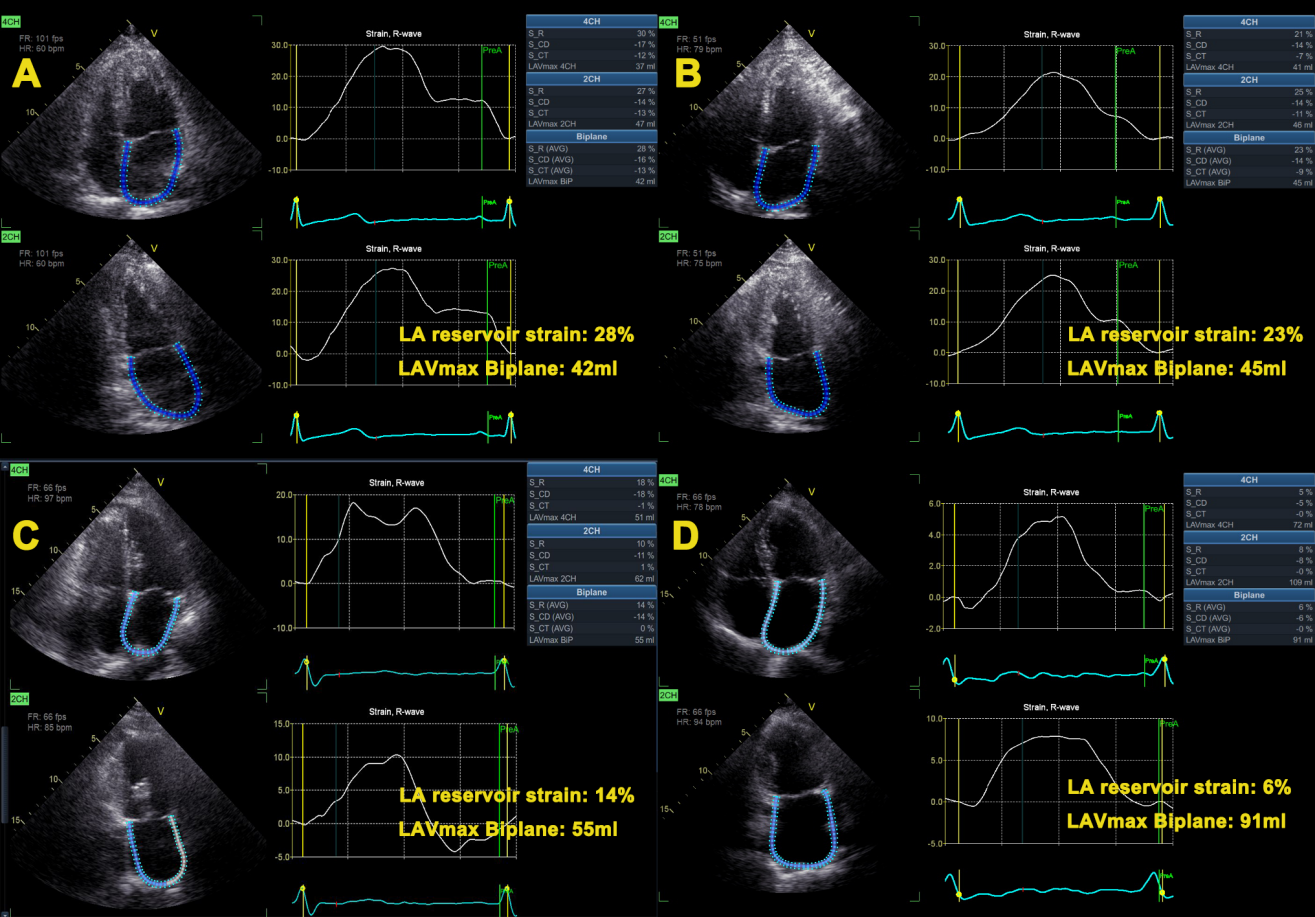
Representative cases. Left atrial strain curves of subjects with sinus rhythm (**A**), paroxysmal atrial fibrillation without left atrial enlargement (**B**), and persistent atrial fibrillation without (**C**) and with (**D**) left atrial volume enlargement. From A to D, the LA reservoir strain gradually decreased. LA, left atrial; LAVmax, left atrial maximal volume.

LA function consists of three components, namely, reservoir, conduit, and active pump. The total function of the LA is best reflected by reservoir strain corresponding to LA early diastole with maximum relaxation of its wall, algebraically positive. LA strain was evaluated using AFI-LA methods by the R-wave gating from the apical two- and four-chamber views. During the processing, the LA endocardium surface is manually traced by a point-and-click approach. The epicardial surface tracing is automatically generated by the system in order to obtain a region of interest (ROI). The ROI definition usually starts with delineating the endocardial contour, which should be drawn from the mitral annulus on one side, extrapolates across the pulmonary vein and/or LA appendage orifices, and ends at the mitral annulus on the opposite side. The ROI can be manually adjusted in width and shape, and then the software automatically tracks the quality for each segment and gives the peak LA reservoir strain and strain curves (18) (Figure 1).

LV strain was measured using the AFI method from the apical two-, three-, and four-chamber views (19). The software analyzed the myocardial motion by tracking frame-to-frame speckle changes. When necessary, automatic endocardial recognition was manually adjusted to ensure correct “anchorage” of the algorithm to the mitral annulus, exclude papillary muscles and chordae from tracking, and correctly include the LV apex. The ROI was eventually adjusted to ensure tracking of the whole myocardial thickness. LV outflow pulsed Doppler was used to time end systole. The segmental strain curves in apical view and 18-segment bull's-eye diagrams related to strain parameters were automatically displayed. GLS was calculated as the average value of the peak systolic strain in 18 LV myocardial segments. All strain measurements were conducted in accordance with the EACVI/ASE/Industry Task Force guidelines (17, 18).

### 2.5. Statistical analysis

All statistical analyses were conducted using R version 4.1.1 (R Foundation for Statistical Computing, Vienna, Austria). Normally distributed continuous data were expressed as mean ± standard deviation, while non-normally distributed data were expressed as median and interquartile range (IQR). Normality distribution was checked using the Shapiro–Wilk test and Q–Q plots. The differences among groups were analyzed using the one-way analysis of variation (ANOVA) for normally distributed data with the Bonferroni correction for pairwise comparisons between the two groups. The Kruskal–Wallis rank sum test was used for non-normally distributed data, and the all-pairwise method was used for further pairwise comparisons between the two groups. Categorical data were presented as percentages and analyzed using the *χ*^2^-test or Fisher's exact test as appropriate. A receiver operator characteristic (ROC) was performed to obtain the areas under the curve (AUC) and 95% confidence intervals (CIs). A two-tailed *P*-value < 0.05 was considered a statistically significant difference.

## 3. Results

### 3.1. Clinical and echocardiographic characteristics stratified by controls and AF subtype

**Figure 2:**
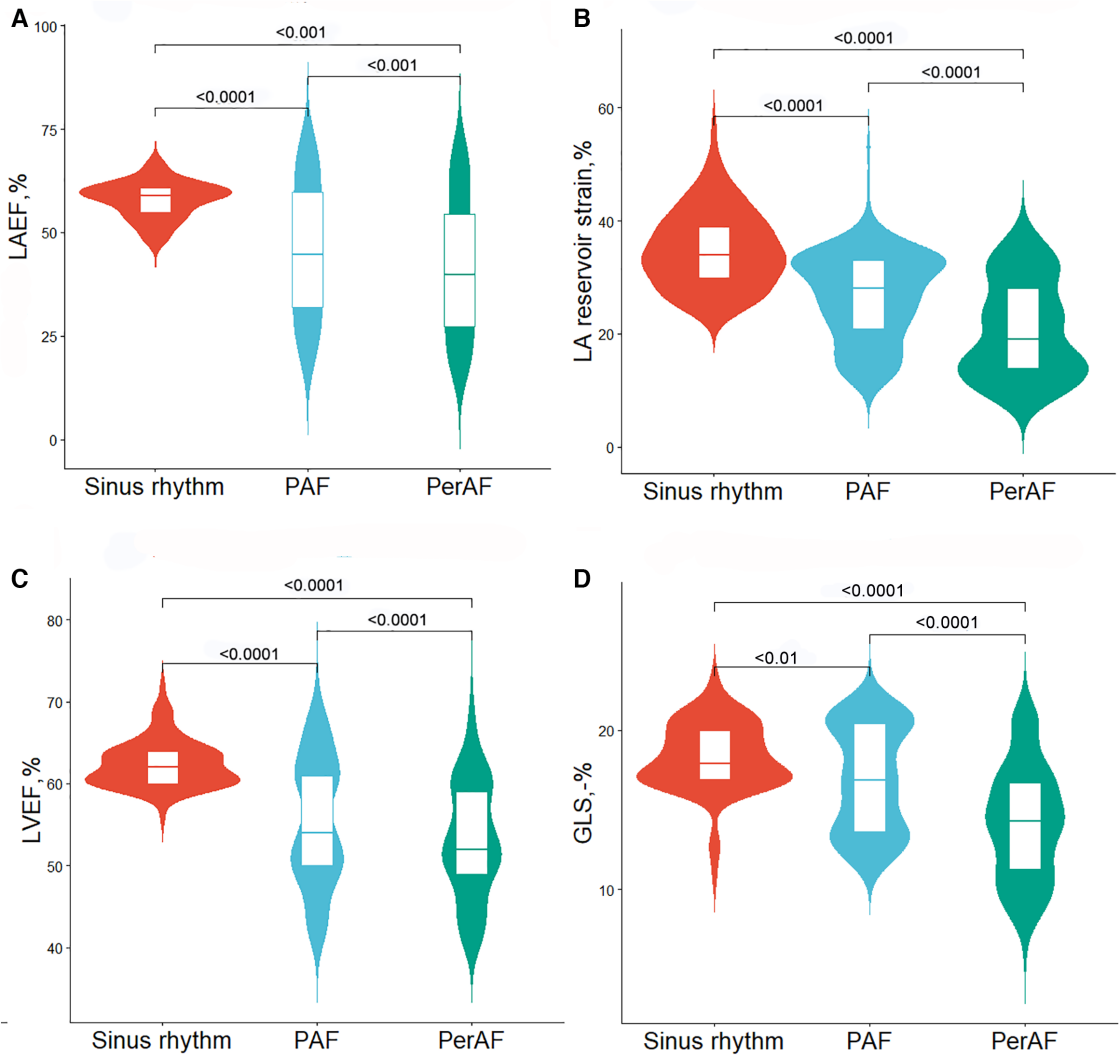
Violin plots for comparisons of echocardiographic characteristics between controls and atrial fibrillation subtype. (**A**) LAEF, (**B**) LA reservoir strain, (**C**) LVEF, and (**D**) GLS. PAF, paroxysmal atrial fibrillation; PerAF, persistent atrial fibrillation; LAEF, left atrial emptying fraction; LA, left atrial; LVEF, left ventricular ejection fraction; GLS, global longitudinal strain. Wider sections of the violin plot represent a higher distribution probability, the thin line in the center white box represents the median, and the white box in the center of the violin represents the interquartile range.

Patient characteristics stratified by controls and AF subtype are summarized in Table 1 and Figure 2. Compared with the sinus rhythm group, both AF groups showed higher CHA_2_DS_2_-VASc score, blood pressure, and heart rate, with a higher proportion of hypertension and EHRA classes 3–4. Moreover, both AF groups had greater LA dimensions, dilated LA maximum and minimum volumes, and volume indexes than those in the sinus rhythm group. In addition to LA structure, LA function was impaired in both AF groups, showing decreased LA emptying fraction (LAEF) and impaired LA reservoir strain than sinus rhythm (Graphical Abstract).

**Table 1:** Clinical and echocardiographic characteristics in controls (sinus rhythm) and atrial fibrillation subtype.

Variables	Sinus rhythm (*n* = 102)	PAF (*n* = 284)	PerAF (*n* = 179)	*P*-value
Clinical characteristics				
Age, years	64 ± 10	63 ± 12	64 ± 11	0.84
Male, *n* (%)	65 (64)	187 (66)	113 (63)	0.82
Body mass index, kg/m^2^	23.4 ± 2.5	24.1 ± 3.6	24.1 ± 3.3	0.14
Systolic blood pressure, mm Hg	121 ± 8	131 ± 20^a^	130 ± 19^a^	<0.001
Diastolic blood pressure, mm Hg	74 ± 9	81 ± 13^a^	80 ± 12^a^	<0.001
Heart rate, bpm	72 ± 9	81 ± 17^a^	78 ± 15^a^	<0.001
CHA_2_DS_2_-VASc score	0.4 ± 0.8	0.9 ± 0.4^a^	0.9 ± 0.5^a^	<0.001
EHRA classes 3–4, *n* (%)	0 (0.0)	61 (22)^a^	44 (25)^a^	<0.001
Current smoking, *n* (%)	10 (10)	44 (16)	31 (17)	0.23
Hypertension, *n* (%)	10 (10)	120 (42)^a^	76 (43)^a^	<0.001
Diabetes, *n* (%)	8 (8)	30 (11)	21 (12)	0.59
Dyslipidemia, *n* (%)	3 (3)	24 (9)	12 (7)	0.17
Medication				
Antiarrhythmic medication, *n* (%)		134 (47)	84 (47)	0.84
Beta-blockers, *n* (%)		98 (35)	61 (34)	0.97
Calcium blocker, *n* (%)		33 (12)	21 (12)	0.92
Anticoagulation, *n* (%)		185 (65)	114 (64)	0.83
Echocardiographic parameters				
LA dimension, mm	32 ± 2	42 ± 7^a^	43 ± 7^a^	<0.001
LA volume—min, ml	21 (20, 22)	39 (27, 59)^a^	44 (28, 67)^a,b^	<0.001
LA volume—max, ml	50 ± 5	77 ± 25^a^	86 ± 36^a,b^	<0.001
LA volume index—min, ml/m^2^	13 (12, 13)	20 (13, 31)^a^	23 (14, 38)^a,b^	<0.001
LA volume index—max, ml/m^2^	30 ± 3	45 ± 15^a^	51 ± 22^a,b^	<0.001
LAEF, %	58 ± 4	46 ± 16^a^	41 ± 16^a,b^	<0.001
LA reservoir strain, %	34 (30, 39)	28 (21, 33)^a^	19 (14, 28)^a,b^	<0.001
E/e’	9.9 ± 1.9	11.1 ± 4.6^a^	12.1 ± 4.4^a,b^	<0.001
LV end-diastolic dimension, mm	47 ± 8	46 ± 9	48 ± 3	0.134
LVEDV, ml	86 ± 15	92 ± 24^a^	98 ± 37^a^	0.001
LVESV, ml	31 (29, 35)	37 (32, 48)^a^	42 (33, 54)^a,b^	<0.001
LVEDV index, ml/m^2^	52 ± 9	54 ± 16^a^	59 ± 23^a,b^	0.008
LVESV index, ml/m^2^	19 (17, 22)	22 (18, 28)^a^	25 (19, 32)^a,b^	<0.001
RWT	0.45 ± 0.08	0.47 ± 0.13	0.49 ± 0.12	0.092
LV mass index, g/m^2^	88 (72, 92)	92 (80, 103)	90 (74, 108)	0.069
LVEF, %	62 ± 3	55 ± 9^a^	53 ± 8^a,b^	<0.001
GLS, -%	18.3 ± 2.1	16.9 ± 3.3^a^	14.1 ± 3.5^a,b^	<0.001

Data are presented as mean ± SD or median (interquartile range) for continuous variables and count (%) for categorical variables. PAF, paroxysmal atrial fibrillation; PerAF, persistent atrial fibrillation; CHA_2_DS_2_VASc, history of congestive heart failure, hypertension, diabetes mellitus, stroke/transient ischemic attack/prior thromboembolism, vascular disease, age, and sex; EHRA, European Heart Rhythm Association; LA, left atrial; min: minimum; max: maximum; LAEF, left atrial emptying fraction; E/e’, mitral inflow peak early diastolic velocity/mitral annular peak early diastolic velocity; LV, left ventricular; RWT, relative wall thickness; LVEDV, left ventricular end-diastolic volume; LVESV, left ventricular end-systolic volume; LVEF, left ventricular ejection fraction; GLS, global longitudinal strain.

^a^ *P* < 0.05 vs. the sinus rhythm group.

^b^ *P* < 0.05 vs. the paroxysmal atrial fibrillation group.

In terms of LV, both AF groups showed greater LV end-diastolic volume (LVEDV) and LV end-systolic volume (LVESV), higher mitral inflow peak early diastolic velocity/mitral annular peak early diastolic velocity (E/e'), and impaired LV systolic function compared to those in the sinus rhythm group.

The PerAF group showed dilated LA maximum and minimum volume, decreased LAEF, and impaired LA reservoir strain than those in the PAF group. A higher LVESV, LVESV index, and E/e' and lower LVEF and absolute GLS value were also shown in the PerAF group than those in the PAF group.

### 3.2. Cardiac structure and function remodeling in AF patients

**Figure 3:**
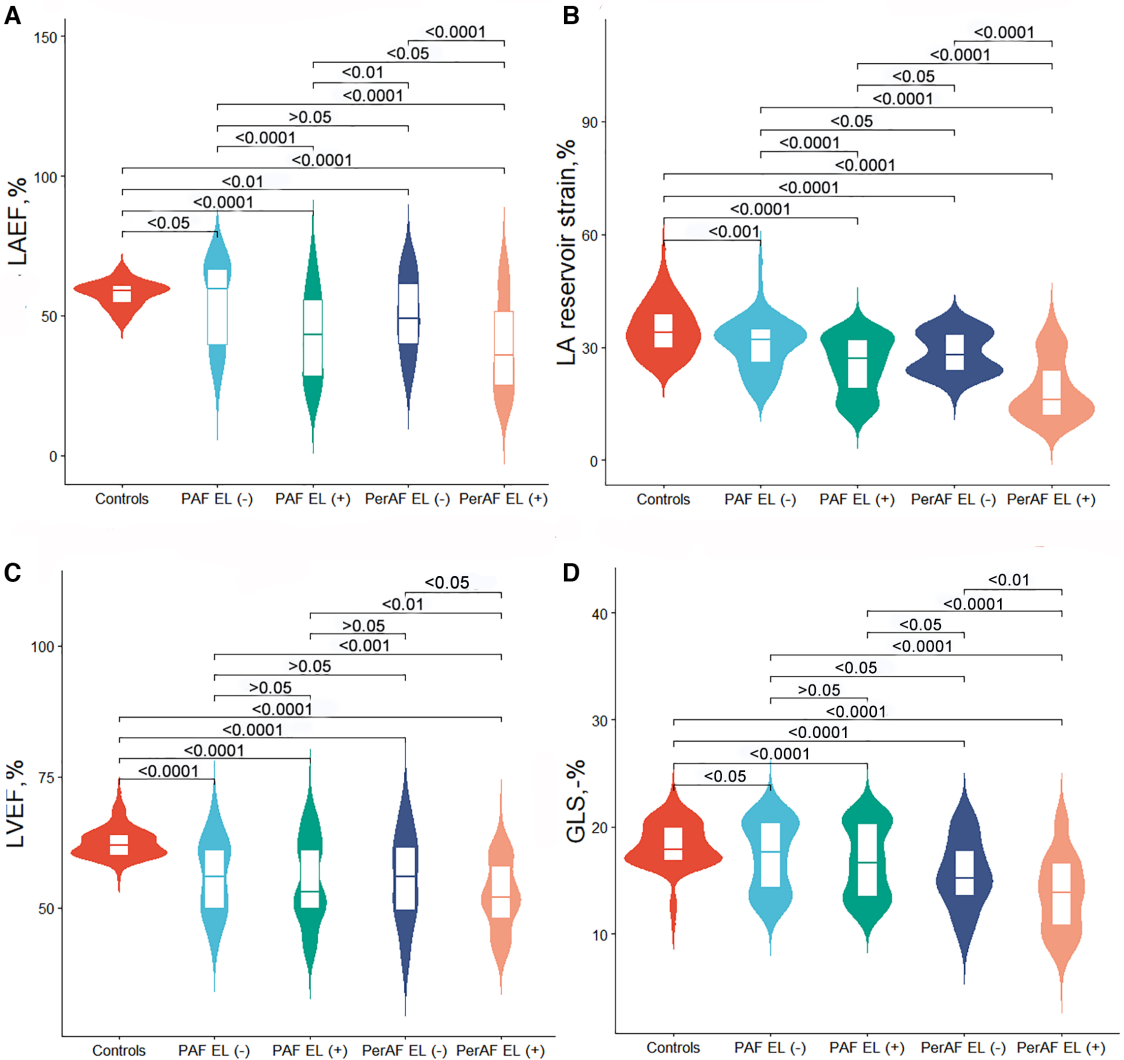
Violin plots for comparison of echocardiographic characteristics between controls and atrial fibrillation subgroup with or without left atrial volume enlargement. (**A**) LAEF, (**B**) LA reservoir strain, (**C**) LVEF, and (**D**) GLS. PAF, paroxysmal atrial fibrillation; PerAF, persistent atrial fibrillation; EL, enlargement; LAEF, left atrial emptying fraction; LA, left atrial; LVEF, left ventricular ejection fraction; GLS, global longitudinal strain.

To gain insight into the association between LA size and function, both AF groups were dichotomized into groups according to the recommended LA maximum volume index (≤34 ml/m^2^) (20). There were 70 patients with PAF and normal LA maximum volume index [PAF EL (−) group], 214 patients with PAF and dilated LA maximum volume index [PAF EL (+) group], 39 patients with PerAF and normal LA maximum volume index [PerAF EL (−) group], and 140 patients with PerAF and dilated LA maximum volume index [PerAF EL (+) group]. Compared with the sinus rhythm group, patients with AF regardless of LA enlargement had significantly lower LAEF, LA reservoir strain, LVEF, and GLS, indicating that impairment of LA and LV function occurred before LA enlargement (Graphical Abstract, Table 2 and Figure 3).

**Table 2:** Echocardiographic characteristics stratified according to atrial fibrillation subgroups with or without left atrial enlargement.

Variables	Sinus rhythm (*n* = 102)	PAF EL (−) (*n* = 70)	PAF EL (+) (*n* = 214)	PerAF EL (−) (*n* = 39)	PerAF EL (+) (*n* = 140)	*P*-value
LAEF, %	58 ± 4	55 ± 15^a^	43 ± 16^a,b^	51 ± 13^a,c^	39 ± 16^a,b,c,d^	<0.001
LA reservoir strain, %	34 (30, 39)	32 (26, 35)^a^	27 (19, 32)^a,b^	28 (24, 34)^a,b,c^	16 (12, 24)^a,b,c,d^	<0.001
LVEDV, ml	86 ± 15	87 ± 15	94 ± 26^a,b^	89 ± 22^c^	101 ± 39^a,b,c,d^	<0.001
LVESV, ml	31 (29, 35)	36 (33, 43)^a^	38 (32, 50)^a^	38 (33, 46)^a^	44 (33, 56)^a,b^	<0.001
LVEDV index, ml/m^2^	52 ± 9	52 ± 12	55 ± 17^a,b^	55 ± 15^a,b^	60 ± 24^a,b,c,d^	0.008
LVESV index, ml/m^2^	19 (17, 22)	21 (18, 26)^a^	22 (18, 29)^a^	24 (20, 29)^a,b,c^	26 (19, 33)^a,b,c,d^	<0.001
E/e’	9.9 ± 1.9	8.7 ± 2.8^a^	11.9 ± 4.8^a,b^	11.4 ± 4.4^b^	12.4 ± 4.4^a,b^	<0.001
LVEF, %	62 ± 3	56 ± 7^a^	55 ± 10^a^	56 ± 8^a^	53 ± 7^a,b,c,d^	<0.001
GLS, -%	18.3 ± 2.1	17.3 ± 3.2^a^	16.8 ± 3.3^a^	15.6 ± 3.2^a,b,c^	13.7 ± 3.5^a,b,c,d^	<0.001

Data are presented as mean ± SD or median (interquartile range) for continuous variables. PAF, paroxysmal atrial fibrillation; PerAF, persistent atrial fibrillation; EL, enlargement; LAEF, left atrial emptying fraction; LA, left atrial; LVEDV, left ventricular end-diastolic volume; LVESV, left ventricular end-systolic volume; E/e’, mitral inflow peak early velocity/mitral annular peak early velocity; LVEF, left ventricular ejection fraction; GLS, global longitudinal strain.

^a^ *P* < 0.05 vs. the sinus rhythm group.

^b^ *P* < 0.05 vs. PAF EL (−) group.

^c^ *P* < 0.05 vs. PAF EL (+) group.

^d^ *P* < 0.05 vs. PerAF EL (−) group.

In patients with PAF, LAEF and LA reservoir strain were lower in patients with LA enlargement than those in patients without LA enlargement. Similarly, in patients with PerAF, LAEF and LA reservoir strain were lower in patients with LA enlargement than those in patients without LA enlargement. These results demonstrated that LA function was further impaired as LA volume expands (Table 2 and Figure 3).

Compared with the PAF EL (+) group, LAEF and LA reservoir strain were lower in the PerAF EL (+) group while higher in the PerAF EL (−) group. Patients with PerAF regardless of LA dilation had higher LVESV index and impaired GLS than those in the PAF EL (+) group. These results demonstrated that as AF progressed, LV systolic function significantly decreased while LA function was also related to the presence of LA enlargement (Table 2 and Figure 3).

**Figure 4:**
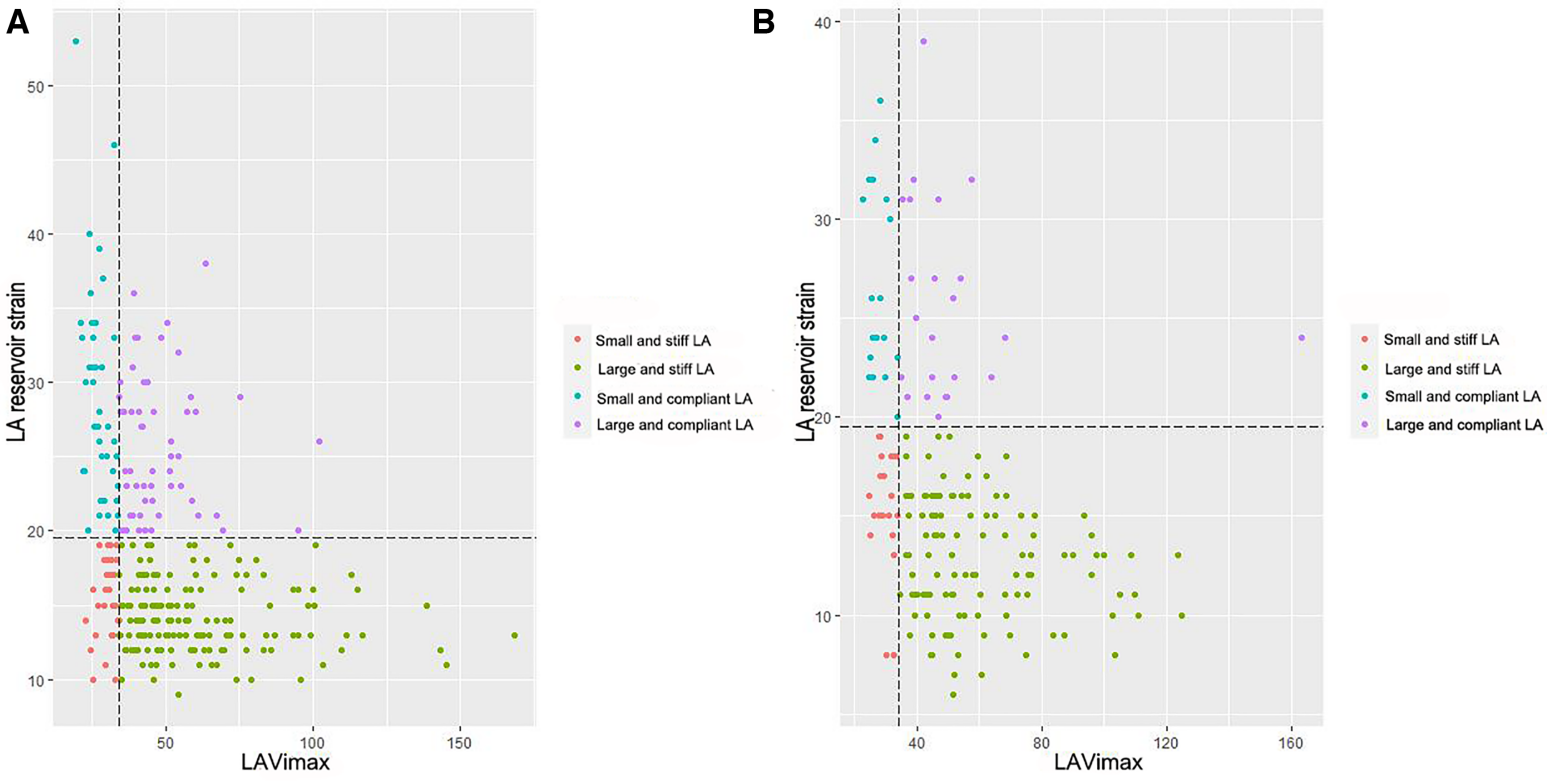
The distribution prevalence of left atrial anatomical remodeling and functional impairment according to atrial fibrillation subtype. (**A**) paroxysmal atrial fibrillation and (**B**) persistent atrial fibrillation. LA, left atrium; LAVimax, left atrial maximal volume index.

To further explore the association between LA structure and function, according to the cutoff of LA reservoir strain obtained from ROC, the LA myocardium was divided into compliant (LA reservoir strain ≥24%) and stiff (LA reservoir strain < 24%). Table 3 and Figure 4 list the prevalence of LA anatomical remodeling and functional impairment according to AF subtype. The PAF group most frequently had large yet compliant LA, whereas the PerAF group most frequently had large and stiff LA (*P* < 0.001). Notably, there were 32 (18.4%) PerAF patients with large but compliant LA and 14 (4.9%) PAF patients and 15 (8.4%) PerAF patients with small but stiff LA, which demonstrated that LA function might be normal even if the size was enlarged, whereas the size might be normal even if the function was impaired.

**Table 3:** Left atrial anatomic remodeling and functional impairment according to the type of atrial fibrillation.

	PAF (*n* = 284)	PerAF (*n* = 179)	*P*-value
Small and compliant LA	56 (19.7)	24 (13.4)	<0.001
Small but stiff LA	14 (4.9)	15 (8.4)	<0.001
Large but compliant LA	123 (43.3)	32 (18.4)	<0.001
Large and stiff LA	91 (32.0)	107 (59.8)	<0.001

PAF, paroxysmal atrial fibrillation; PerAF, persistent atrial fibrillation; LA, left atrial.

### 3.3. Evaluation of echocardiographic parameters in the detection of AF

**Figure 5:**
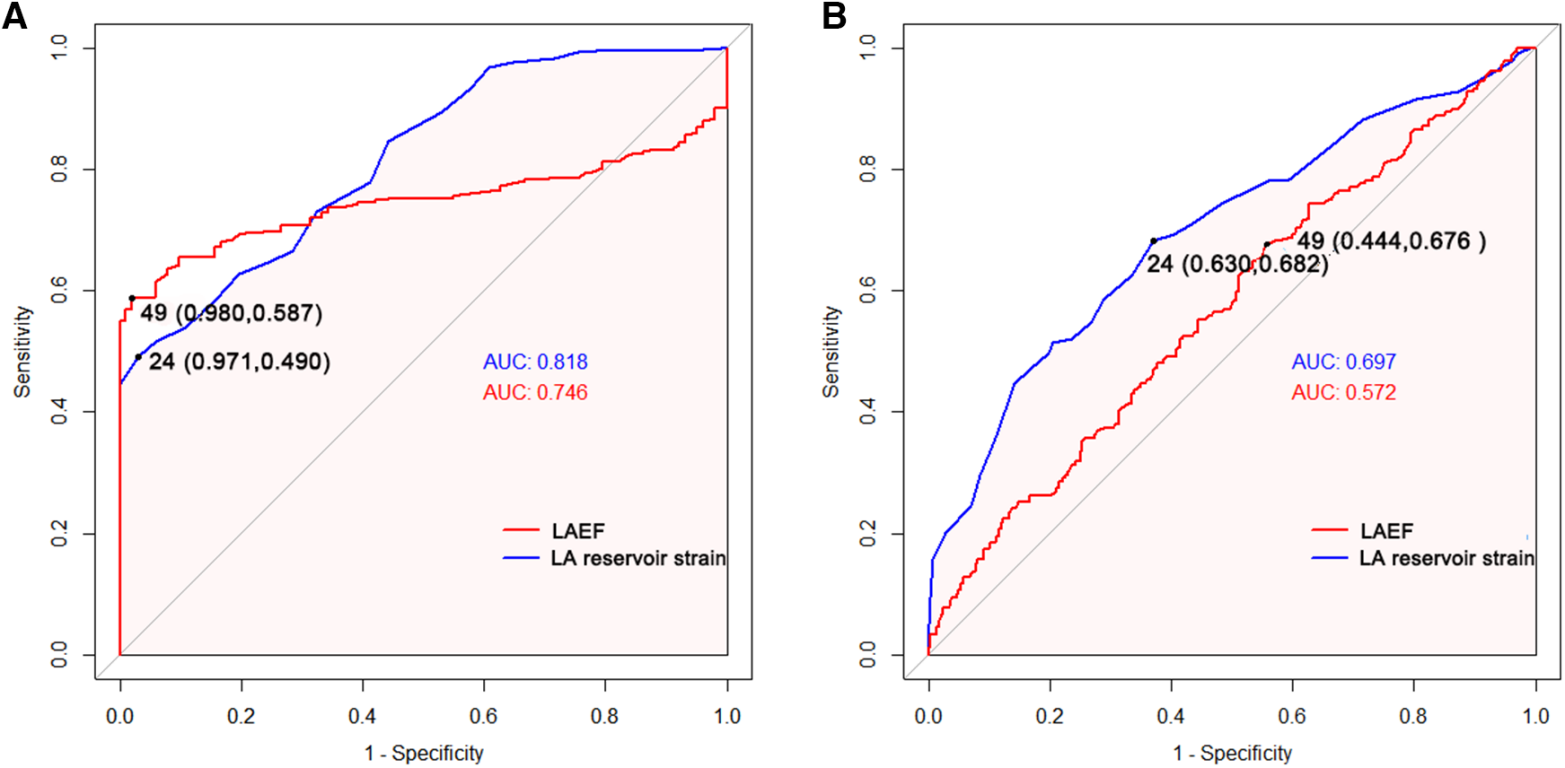
Receiver operating characteristic curves of LA function parameters to identify atrial fibrillation in all patients (**A**) and persistent atrial fibrillation in all atrial fibrillation patients. (**B**) AUC, area under the curve; LAEF, left atrial emptying fraction; LA, left atrial.

ROC curve analyses showed that LA reservoir strain had a relatively higher diagnostic value than LAEF not only in distinguishing between AF and sinus rhythm (AUC: LA reservoir strain vs. LAEF 0.82 vs. 0.75) but also in distinguishing between PAF and PerAF (AUC: LA reservoir strain vs. LAEF 0.70 vs. 0.57) (Figure 5).

## 4. Discussion

The present study, which was conducted on 463 patients with AF and 102 subjects with sinus rhythm, identified several important findings. (1) Compared with sinus rhythm, both PAF and PerAF were associated with larger LA and LV volumes, as well as impaired LA and LV function. (2) PerAF had larger LA maximum and minimum volume and more impaired LA and LV function than PAF (3) The impairment of LA and LV function occurred before LA enlargement in AF. (4) The impairment of LA function was significantly aggravated when LA volume was enlarged in AF. (5) As AF progressed, LV systolic function significantly decreased while LA function varied depending on the presence of LA enlargement.

### 4.1. AF-induced changes in LA detected by echocardiography

Consistent with previous studies (21, 22), our study confirmed that AF was significantly associated with LA anatomical and functional remodeling. Additionally, our findings confirmed on a larger scale that AF transition from paroxysmal to persistent was often characterized by advancing atrial structural and functional remodeling, which are in accordance with the findings of Olsen et al. (8).

Among the LA function parameters, our report showed that LA reservoir strain had a higher value than LAEF in distinguishing not only between AF and sinus rhythm but also between PAF and PerAF. Raman et al. (23) had similar results with the magnetic resonance feature-tracking strain, which illustrated that LA reservoir strain was a major predictor of the onset of AF in patients with hypertrophic cardiomyopathy. It might be because the LA reservoir function was the most important feature of AF since it respectively reflected the compliance and loading conditions of LA. The development of AF evolves from sole rhythm disturbance to complex cardiomyopathy (24). Experimental studies have shown that the atrial remodeling of AF is characterized by the presence of predominantly interstitial fibrosis, which impacts atrial compliance (25, 26). Additionally, interstitial fibrosis promotes replacement fibrosis, resulting in impaired contractile function of LA cardiomyocytes. Therefore, a comprehensive assessment of LA volume and reservoir strain may provide additional insight into LA remodeling caused by AF.

### 4.2. LA volume and function remodeling were not always concordant in AF

In line with the study previously mentioned (10), the present report showed that LA volume and function were not always concordant in AF. LA function remodeling has been already impaired before LA volume enlargement, whereas even with enlarged LA volume, LA function impairment may not be apparent. The study also highlighted that large yet compliant atria were more prevalent in PAF, which may indicate a more advanced stage of disease with atrial enlargement yet without impaired strain. It is reported that this condition could have a greater likelihood of successful AF ablation (27).

The study also indicated that LA function was severely impaired when LA volume was enlarged in AF. This could be because the increased LA volume can increase wall stress, triggering myocyte hypertrophy and fibrosis (28). In addition, LA reservoir strain was higher in PerAF without dilated LA volume than that in PAF with dilated LA, suggesting that LA reservoir strain was not only associated with the AF subtype but also related to the LA volume enlargement.

Although there are several papers demonstrating the impact of AF on LA remodeling as well as the utility of LA size and function on AF recurrence prediction (29–31), our study shows that patients with PerAF had a higher degree of LA anatomical remodeling and functional impairment than that in patients with PAF, reflecting different stages of the disease. Moreover, LA reservoir strain could be a useful indicator in estimating cardiac function remodeling induced by AF even before LA volume dilation and showed better discriminative value than LAEF to separate AF from sinus rhythm patients. This is important to facilitate appropriate clinical management decisions, because both anatomical remodeling and functional impairment could have implications for the success of AF ablation. Therefore, the added value of LA strain detected by speckle tracking echocardiography allows effective triaging of the AF patient, suggesting that it should be implemented in the systematic evaluation of AF patients before ablation. The ability to identify mild disease before morphological changes provides a basis for risk stratification before surgery, particularly in patients with non-dilated LA, and allows more selective prophylactic therapy for postoperative complications. According to Ma et al. (32), LA strain could be of great use in identifying patients with a high risk of AF recurrence after catheter ablation. It is reported that LA strain predicted the incidence of post-operative AF independently of LA dilation in severe aortic stenosis (33). It has also been proven to provide a diagnostic role of thrombotic risk assessment for non-valvular AF patients planned for electrical cardioversion (34). Additionally, patients who have suffered from specific cardiomyopathies and valve diseases such as myocardial infarction are at risk of developing AF, which may lead to a prognostic value of LA strain as a significant predictor of incident AF (35). Nevertheless, there are several limitations of LA strain including the lack of a “universal” definition of normal ranges and the impact of vendors/segmentation on data reproducibility. Although LA strain consists of three phases, i.e., reservoir, conduit, and pump, in the case of normal diastolic function, the relative contribution of the particular LA phases into the LV filling is as follows: reservoir, 40%; conduit, 35%; and pump 25% (36). The impaired phasic function of the LA was described in many cardiovascular diseases. However, in patients with PerAF at the time of the echocardiographic exam, it is impossible to measure the LA pump strain. Further studies are needed to focus on LA strain analysis, especially the reservoir strain, with a large sample to analyze its value in clinical practice.

### 4.3. Left atrioventricular functional coupling across different stages of AF progression

In our study, LV systolic and diastolic function was significantly impaired in AF than sinus rhythm, which was consistent with the study by Ross Agner et al. (37), who reported that GLS was significantly impaired in AF compared to sinus rhythm controls independent of age, sex, heart rate, LVEF, and LV mass. Our study also suggested that subclinical alterations in LV function may have preceded the deterioration of LV volume dilation.

We found that patients with AF had significantly higher heart rates compared to those in patients with sinus rhythm. However, the fact that myocardial oxygen consumption increases and myocardial efficiency decreases with increased heartbeat is well known. Literature on the relationship between heart rate and strain measurements showed that GLS in normal subjects with high and low heart rates was similar (38). All strain and strain rate variables in the longitudinal, circumferential, and radial directions were not significantly different between pacing rates (39).

Previous studies have shown that atrioventricular coupling is a dynamic and time-variant process in AF (40). On the one hand, AF can lead to progressive ventricular remodeling through tachycardia and irregular ventricular rhythm (41). On the other hand, LV fibrosis in patients with AF may reflect the same process in the LV that leads to atrial fibrosis, which may be associated with a pathologic myocardial process that triggers AF recurrence (40). It is reported that LA reservoir function is closely associated with heart failure (42). AF-associated ventricular remodeling results in myocardial fibrosis, chamber dilation, and mitral and tricuspid regurgitation, all of which contribute to damaging LV diastolic as well as systolic function (43).

## 5. Limitations

There were several limitations in the present study. First, software available on the market used different algorithms of strain analysis with possible consequent biases in comparison between studies. Second, the number of samples, especially sinus rhythm controls (*n* = 102) and PerAF patients without LA dilation (*n* = 39), was small, and more patients would be required to provide a robust conclusion in this aspect. Third, there was a lack of comparison with other imaging techniques such as three-dimensional echocardiography and cardiac magnetic resonance imaging. Forth, this is a transversal study, and we cannot conclude that LA strain detects an early disease but, rather, that LA strain diagnoses a mild disease. Additionally, there are various factors affecting LA strain. These factors change simultaneously in most cases, making it difficult to assess the net influence of each factor. Through the transversal study, we cannot determine whether the causes of the decrease of LA function and increase of LA volume in the population of patients with AF are the morpho-pathological changes at the level of the LA caused by the presence of AF or if they are the cause of AF. These confounding factors might also have biased the results. A prospective study in a larger patient population is required to clarify the influence of these confounding factors on LA strain in patients with AF and validate the predictive value of LA strain on long-term outcomes.

## 6. Conclusions

Compared with the normal sinus rhythm group, patients with AF had dilated LA volume and impaired LA function, which were further worsened in patients with PerAF than those in patients with PAF. LA volume and function remodeling were not always concordant in AF, and LA function impairment could occur before LA volume enlargement. Left atrioventricular remodeling happened across different stages of AF development, and patients with AF had significantly more impaired GLS than the normal sinus rhythm group.

## Data Availability

The raw data supporting the conclusions of this article will be made available by the authors, without undue reservation.
